# Genetic Identification of the Wild Form of Olive (*Olea europaea var. sylvestris*) Using Allele-Specific Real-Time PCR

**DOI:** 10.3390/foods9040467

**Published:** 2020-04-09

**Authors:** Christina I. Kyriakopoulou, Despina P. Kalogianni

**Affiliations:** Department of Chemistry, University of Patras, 26504 Rio Patras, Greece; xrkyriako@gmail.com

**Keywords:** *Olea europaea var Sylvestris*, oleaster, olive, olive oil, real-time PCR, adulteration, SNP, DNA

## Abstract

The wild-type of olive tree, *Olea europaea var Sylvestris* or oleaster, is the ancestor of the cultivated olive tree. Wild-type olive oil is considered to be more nutritious with increased antioxidant activity compared to the common cultivated type (*Olea europaea* L. *var Europaea*). This has led to the wild-type of olive oil having a much higher financial value. Thus, wild olive oil is one of the most susceptible agricultural food products to adulteration with other olive oils of lower nutritional and economical value. As cultivated and wild-type olives have similar phenotypes, there is a need to establish analytical methods to distinguish the two plant species. In this work, a new method has been developed which is able to distinguish *Olea europaea var Sylvestris* (wild-type olive) from *Olea europaea* L. *var Europaea* (cultivated olive). The method is based, for the first time, on the genotyping, by allele-specific, real-time PCR, of a single nucleotide polymorphism (SNP) present in the two olives’ chloroplastic genomes. With the proposed method, we were able to detect as little as 1% content of the wild-type olive in binary DNA mixtures of the two olive species.

## 1. Introduction

The wild form of the olive tree, formally named *Olea europaea var Sylvestris* or oleaster, is considered to be one of the oldest trees worldwide; it is found mainly in the Mediterranean Basin. Genetic pattering studies have shown that cultivated olive trees, i.e., *Olea europaea* L. *var Europaea*, are more similar to oleaster species, providing evidence to support the concept that oleasters are the ancestors of cultivated trees [1]. Both wild and cultivated olive oil have beneficial properties for human health, giving them high economic and nutritional value; however, this has made olive oil one of the most vulnerable agricultural products to fraud and fakery. Wild-type olive oil has higher antioxidant activity, as well as phenolic, tocopherolic and orthodiphenolic contents equal to or higher those in extra virgin cultivated olive oil [2]. Moreover, wild-type olive is a valuable natural resource due to its resistance to certain environmental and climatic conditions and diseases [3]. For the above reasons, its genetic characteristics have to be evaluated, and reliable molecular tools have to be developed for olive oil origin traceability (genetically and geographically) and wild-type olive oil identification. On the other hand, producers need accurate analytical tools for the genetic identification of their wild-type olive-related products to ensure their high added value [4].

Genetic variations between the two plant species have not been extensively explored by the research community. The analytical techniques used so far for the genetic identification of the wild form of olive tree include randomly amplified polymorphic DNA (RAPD), amplified fragment length polymorphisms (AFLPs) and intersimple and simple sequence repeats (ISSRs and SSRs), based on the chloroplastic and mitochondrial plant DNA [1]. Early research compared the genome of *Olea europaea* L. *var Europaea* to that of the wild-type olive, derived from many countries and two areas in Italy, using AFLP analysis as designed by Angiolillo et al., 1999, and Baldoni et al., 2006 [5,6]. RAPD analysis was used to distinguish oleasters from *Olea europaea* L. *var Europaea* trees on the Mediterranean islands of Corsica and Sardinia, as well as in Turkey [7,8]. Besnard et al. used RAPD markers and restriction fragment length polymorphism (RFLP) analysis based on mitochondrial and cytoplasmatic DNA to investigate the relationships among olive species and subspecies in the Mediterranean Basin and other countries in Asia and Africa. This research led to the discovery that there was a large degree of diversity among olive cultivated trees, but that they were more or less related to the local oleasters [9,10]. Moreover, ISSR and SSR markers have been utilized by many researchers to investigate the relation and differentiation of cultivated olives from wild-type olives [3,11,12,13,14,15,16]. Genome size estimation based on double-stranded DNA staining followed by flow cytometric analysis was also used for screening purposes between *Olea europaea var Sylvestris* and *Olea europaea* L. *var Europaea* species [17], while flow cytometry in combination with SSR profiles was used for the taxonomy of four olive subspecies, namely *Olea europaea ssp. cerasiformis*, *Olea europaea ssp. guanchica*, *Olea europaea var Sylvestris* and *Olea europaea* L. *var Europaea* [18].

Moreover, the wild olive has also been used for nonedible purposes in pharmacology and cosmetics to create products with specific valuable characteristics. Researches have also studied the antimicrobial activity of the wild olive against certain human bacterial pathogens [19]. Several plants, including the olive and its wild form, have also been used for the production of various food supplements [20]. Finally, phenolic extracts from wild olive leaves have been investigated for use in foodstuffs, food additives and functional food materials, due to their high antioxidant activity [21,22].

In 2017, the complete genome sequence of *Olea europaea var Sylvestris* was published by Unver et al. [23]. This will be useful, in the future, for the localization of specific genetic variations in the genome of oleasters compared to other olive subspecies.

For the first time, in this work, a single nucleotide polymorphism (SNP)-based method was developed for the detection and identification of the wild form of olive in order to distinguish it from the cultivated olive. Different olive cultivars contain different SNPs in their genome that are responsible for their unique phenotyping characteristics [24,25]. The method was based on an allele-specific, real-time PCR. The proposed method is able to detect wild-type olive DNA at levels as low as 1% in DNA derived from the cultivated olive.

## 2. Materials and Methods

### 2.1. Materials and Instrumentation

The Vent (exo-) DNA polymerase was purchased by New England Biolabs (Beverly, MA, USA). Deoxynucleoside triphosphates (dNTPs) were obtained from Kapa Biosystems (Wilmington, MA, USA). The fluorescent dye SYBR Green I 10^4^ × concentrated was from Molecular Probes (Eugene, OR, USA). The primers used were from Eurofins Scientific (Brussels, Belgium) and are listed in Table 1. The size of the PCR products was 136 bp. An extra virgin olive oil sample (*Olea europaea* L. *var Europaea*) was purchased from a local market, while a certified wild-type olive oil sample (*Olea europaea var Sylvestris*) was kindly by local producer, Alexandros Karakikes, from the Olea Sylvestris estate (Agrielaio, Volos, Greece) [26].

Real-time PCR was performed using the Mini Opticon Real-Time PCR System from Biorad (Hercules, CA, USA), while the results were analyzed using the Bio–Rad CFX Manager 3.0 software.

### 2.2. DNA Isolation Procedure

DNA was isolated from olive oil samples using the NucleoSpin Tissue kit from Macherey-Nagel (Düren, Germany) according to the manufacturer’s instructions. The quantity and purity of the isolated DNA were determined using the Nanodrop UV/VIS Nanophotometer by Implen GmbH (Münich, Germany).

### 2.3. Design of the Primers

The primers used for the amplification of *Olea europaea var Sylvestris* (wild-type olive) and *var Europaea* (cultivated olive) were designed using the free online Oligo Analyzer software for primer evaluation (created by Dr. Teemu Kuulasmaa), based on the *Olea europaea var. sylvestris* NADH dehydrogenase subunit F gene, chloroplastic sequence (Accession Number: AY172114) and the *Olea europaea* L. NADH dehydrogenase subunit F (ndhF) gene chloroplastic sequence (Accession Number: DQ673278) [23].

### 2.4. Allele-Specific, Real-Time PCR

The allele-specific, real-time PCR reactions were conducted in a final volume of 50 μL and contained 1 × Thermopol Buffer (20 mM Tris-HCl, 10 mM (NH_4_)_2_SO_4_, 10 mM KCl, 0.1% Triton^®^ X-100 at pH 8.8), 0.5 μM of each of the upstream and downstream primers, 0.2 mM of each of the four dNTPs, 0.5 mM MgCl_2_, 2 × SYBR Green I, one unit of Vent (exo-) DNA polymerase and 150 ng of isolated DNA. The reaction conditions involved a 95 °C incubation step for three min, followed by 45 cycles at 95 °C for 30 s, 62 °C for 30 s, 72 °C for 30 s and a final extension step at 72 °C for 10 min.

## 3. Results and Discussion

**Figure 1:**
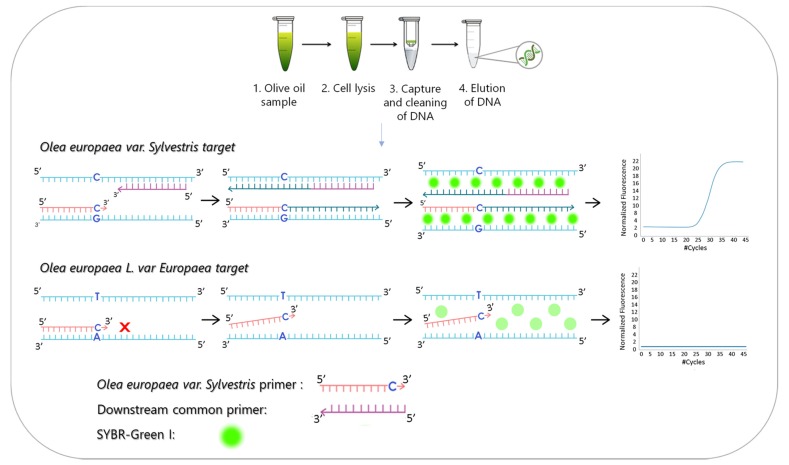
(**Upper panel**) Schematic illustration of the principle of the method that includes DNA isolation and purification from olive oil samples using spin cleanup columns, including the following steps: cell lysis of an olive oil sample, capture of DNA to the cleanup columns and elution of the DNA from the columns. (**Lower panel**) The allele-specific, real-time PCR. Two allele-specific upstream primers that contain the SNP of interest at their 3′ ends and one common downstream primer were used in the amplification reaction. Only the perfectly complementary upstream primer to the target was extended by the DNA polymerase, while the amplicons were detected by the DNA intercalating dye, SYBR Green I.

A new analytical method was developed for the detection and identification of *Olea europaea var Sylvestris* that refers to the wild form of the olive tree. The method was based on the detection of a specific Single Nucleotide Polymorphism (SNP) that is different in the genome of the wild olive plant. The method involves the following steps: (i) DNA isolation from olive oil samples and (ii) allele-specific, real-time PCR using an upstream primer specific to *Olea europaea var Sylvestris* or *var Europaea* species and a common downstream primer. The species-specific primers have the same 22-base sequence but differ only at the base at the 3′ end that contains the SNP of interest. The DNA sequences were amplified using a DNA polymerase that lacked the 3′ to 5′ exonuclease activity, so only the primer that was perfectly complementary to the DNA target was extended by the enzyme. The amplicons were finally detected using the DNA intercalating fluorescent dye SYBR Green I. The principle of the proposed method is illustrated in Figure 1. SYBR Green I was chosen here instead of Taqman probes in order to develop a new analytical method that could be easily transferred, with few modifications, for the detection of other SNPs that will be found in the wild olive genome in the future.

### 3.1. DNA Isolation

First, DNA was isolated from olive oil samples and its concentration was determined using a UV/VIS nanophotometer. It was found that the isolation procedure did not result in a constant DNA amount for all samples, with the DNA concentrations ranging from 8.4 to 142 ng/μL. To avoid fluctuation in the PCR yield due to different initial DNA concentrations, we decided to use the same amount (ng) of isolated DNA for all samples into the real-time PCR mixture. After amplification, the amplicons had a size of 136 bp. The quality of the isolated DNA was also determined by UV measurements; the ratios A_260_/A_280_ were from 1174 to 1739. DNA was considered to be of high quality when the ratio A_260_/A_280_ was above 1.8.

### 3.2. Optimization of the PCR Conditions

The real-time PCR conditions were initially optimized. The parameters studied were the amount of the isolated DNA, the concentration of the primers, the number of PCR cycles and the temperature of the annealing step of the reaction. At low DNA and primer concentrations, low temperature (55–60 °C) and number of cycles < 45, the PCR was not sufficiently efficient. The yield of the reaction also decreased when a high amount of initial DNA target was used. This may be attributed to the fact that the DNA isolated from olive samples has reduced quality, as it contains high amounts of PCR inhibitors that may inhibit the activity of the DNA polymerase [27]. We also observed that the highest reaction yield and specificity were obtained at an annealing temperature of 62 °C.

### 3.3. Specificity of the Allele-Specific Primers

**Figure 2:**
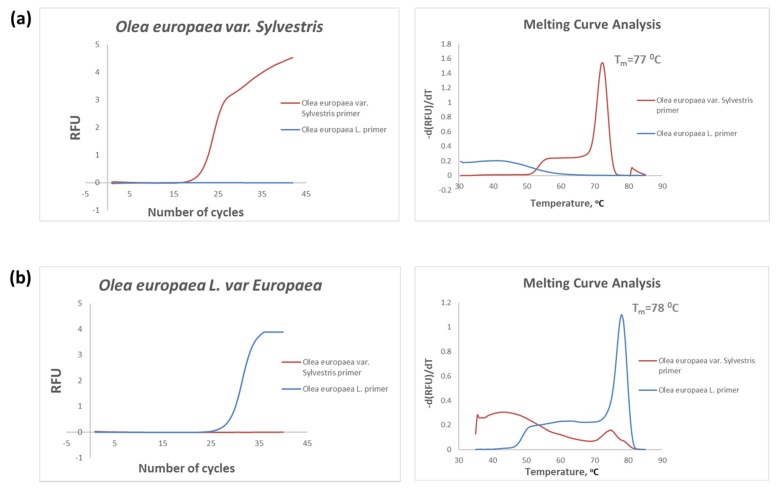
The real-time PCR curves, along with the corresponding melting curve analysis, obtained during the specificity study of the two-allele specific upstream primers with both DNA targets: *Olea europaea var Sylvestris* (wild-type of olive) (**a**) and *Olea europaea* L. *var Europaea* (cultivated olive) (**b**). Each specific primer strictly amplifies the fully complementary DNA sequence. T_m_: melting temperature, RFU: Relative Fluorescence Units.

The specificity of the two species-dependent upstream primers was then studied as follows: both DNA targets, *Olea europaea var Sylvestris* (wild-type olive) and *var Europaea* (cultivated olive) were subjected to two separate amplification reactions using either the upstream primer specific to the wild-type olive or the cultivated olive-specific upstream primer. As shown, in Figure 2, each primer amplified only its fully complementary DNA sequence, proving the superior specificity of the primers. To ensure that the fluorescence signals were attributed only to the specific amplicons, a melting curve analysis was also performed after each amplification reaction. The melting curve analysis revealed only one peak for each PCR product, the melting temperature (T_m_) of which was 77 °C for *Olea europaea var Sylvestris* (wild-type olive) and 78 °C for *var Europaea* (cultivated olive), allowing us to distinguish between the two allele-specific DNA sequences.

### 3.4. Detectability of the Method in Binary DNA Mixtures

**Figure 3:**
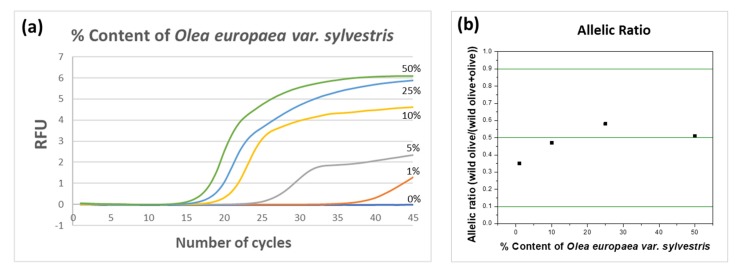
(**a**) The real-time PCR curves obtained for different % DNA content (0–50%) of *Olea europaea var Sylvestris* (wild-type olive) DNA in binary mixtures with *Olea europaea* L. *var Europaea* (cultivated olive) DNA. (**b**) The allelic ratios of the binary DNA mixtures calculated as the ratio of the fluorescence intensity obtained with the upstream primer specific to *Olea europaea var Sylvestris* target versus the sum of the fluorescence intensity obtained by both allele-specific primers for *Olea europaea var Sylvestris* and *var Europaea* targets. All allelic ratios were close to the value of 0.5, which corresponds to a heterozygote sample. RFU: Relative Fluorescence Units.

Subsequently, the detectability of the method in olive DNA binary mixtures was evaluated. DNA mixtures that contained different proportions (1–50%) of DNA from *Olea europaea var Sylvestris* in DNA from *var Europaea* were prepared. An amount of 150 ng of each DNA mixture was then subjected to two separate allele-specific, real-time PCR reactions using each of the species-specific upstream primers along with the common downstream primer, respectively. A high amount of total DNA was used in the PCR in order to detect the low amount of wild olive DNA in the mixtures, e.g., for 150 ng of total DNA in the 1% mixture, only the 1.5 ng was the wild olive DNA. The results are presented in Figure 3. We were able to detect as little as 1% of DNA specific to *Olea europaea var Sylvestris* in the presence of DNA from *Olea europaea* L. *var Europaea*. The allelic ratios of the analyzed SNP for the above DNA mixtures were also calculated based on the fluorescence value at the 45th cycle of the reaction, and are presented in the same Figure. The allelic ratios for all DNA mixtures were close to the value of 0.5, as expected for a heterozygote sample.

### 3.5. Reproducibility of the Method

Finally, the reproducibility of the method was determined. Two different proportions, 1% and 10%, of the above DNA mixtures, were subjected, in triplicate, to real-time PCR. The % coefficients of variation (CV) were calculated based on the obtained Cq values for all samples. The CV for the 1%-content was 10.5% and for the 10%-content was 7.5%, demonstrating the reproducibility of the method.

## 4. Conclusions

A new allele-specific, real-time PCR-based analytical method was developed for the detection and identification of wild-type olive oil (*Olea europaea var Sylvestris*), compared to cultivated olive oil (*Olea europaea* L. *var Europaea*). The discrimination of the two similar plant species was based on genotyping a single nucleotide polymorphism (SNP) that is differently present in the genome of the two plant species. The detection of this SNP was carried out by an allele-specific, real-time PCR that was performed using two different species-specific upstream primers that contained the analyzed SNP and a common downstream primer. Each specific primer amplified only its fully complementary DNA sequence, leading to species identification. The detection of the amplicons was accomplished using the DNA intercalating dye, SYBR Green I. With the proposed method, we were able to sucessfully distinguish between the two plant species in olive oil samples. Also, as little as 1% wild-type olive species was detected in binary DNA mixtures of the two analyzed plant species. In conclusion, the method is easy, rapid, has good detectability, is reproducible and can easily distinguish between species. The proposed method also contributes to the ability to add the higher financial value to wild-type olive-based products. In the future, the determination of different SNPs in the wild-type olive genome compared to all the known cultivated olive trees could lead to more accurate discrimination of the wild-type olive among other olive-based subspecies. The proposed method could also be applied, with some modifications, for the detection of wild olive-based ingredients in food supplements and cosmetic products. The global increase in food supplements has led to the mislabeling of these products and fraudulent practices. In both cases, the purity of the extracted DNA is more important than the PCR yield itself, because several food additives and other ingredients may be present in the extracts, inhibiting the PCR amplification. Also, the amount of the extracted DNA may be extremely low. Thus, the DNA isolation protocols have to be properly justified to remove these inhibitors and increase the DNA recovery and the PCR yield. In some studies, however, the inability to extract DNA from some food supplements has been reported. Finally, in some products, DNA degradation may also occur due to thermal or chemical treatment, but the use of short-length amplicons can overcome this issue [28,29,30,31].

## Figures and Tables

**Table 1:** The primers used in the allele-specific, real-time PCR, two species-specific upstream primers and a common downstream primer, along with their melting temperatures (Tm).

Primer Name	5′–3′ Oligonucleotide Sequence	Melting Temperature *
*Olea europaea var Sylvestris* upstream primer	TGTCAATTTTAATCACTACTGC	62 °C
*Olea europaea* L. *Europaea* upstream primer	TGTCAATTTTAATCACTACTGT	61 °C
Common downstream primer	CTAGTAACTAATCCTAACATGGAA	64 °C

* according to Eurofins Scientific (Brussels, Belgium).
